# Clinical pharmacist interventions in the transition of care in a mental health hospital: case reports focused on the medication reconciliation process

**DOI:** 10.3389/fpsyt.2023.1263464

**Published:** 2023-12-27

**Authors:** Matej Stuhec, Borjanka Batinic

**Affiliations:** ^1^Faculty of Medicine, University of Maribor, Maribor, Slovenia; ^2^Department of Clinical Pharmacy, Ormoz Psychiatric Hospital, Ormoz, Slovenia; ^3^Department of Psychology, Faculty of Philosophy, University of Belgrade, Belgrade, Serbia; ^4^Clinic of Psychiatry, University Clinical Centre of Serbia, Belgrade, Serbia

**Keywords:** psychiatry, hospital clinical pharmacist in psychiatry, transition of care, medication reconciliation, case report

## Abstract

The transition of care represents a key point in the hospital admission and discharge process. A comprehensive transition could lead to fewer medication-related problems. The hospital clinical pharmacist could help in the transition of care process with a comprehensive medication reconciliation process, which has been poorly described in mental health hospitals. This study presents two clinical cases in which hospital clinical pharmacists identified omitted medications and other medication-related issues, including medication errors, during the transition of care in a mental health hospital. These positive experiences may encourage other countries to establish similar collaborations with hospital clinical pharmacists in mental health hospitals.

## Introduction

The transition of patients between different healthcare settings and levels of healthcare is one of the most important therapeutic challenges, as medication errors often occur (1, 2). Medication errors are associated with a higher incidence of adverse effects and higher treatment costs and are responsible for many hospital admissions (1, 2). Medication errors occur at all levels of outpatient and inpatient care. At present, most occur at the admission, ward transition, and discharge from the hospital and back to primary care, so appropriate medication reconciliation is needed (2). The prevalence of medication errors in Scottish hospitals was 7.5% (4,710 patient charts were reviewed) (3). According to the results of the study by Santell et al., 22% of medication errors occur on admission to the hospital, 66% during ward transition, and 12% at the time of discharge, which means that these points are critical in the likelihood of medication errors and need to be particularly highlighted in the healthcare team and effective protective strategies are needed within each healthcare system (4). In a French prospective observational study, of all included all adult patients 29.4% had at least one unintended medication discrepancy, and most were medication errors. Most were omissions (59.3%), and potential severity was observed in almost 40% of patients. The authors proposed different interventions for medication error minimizing, including medication reconciliation, which lead to fewer medication errors (5, 6).

Medication reconciliation is the process of identifying the most accurate list of all medicines a patient is taking, including the data on the name, dosage, frequency, and route of administration of each medicine. Different healthcare professionals could participate in this process (7). Physicians and nurses predominately do medication reconciliation, but hospital clinical pharmacists and pharmacy technicians in some countries also provide medication reconciliation. The medication reconciliation process included five key steps: list the patient’s current medications, list the medications currently needed, compare the lists, make a new list based on the comparison, and communicate the new list to the patient and caregivers (8). In a retrospective chart review study by Smith and Mango (*n* = 720 charts), the researchers investigated the role of the hospital clinical pharmacist in the medication reconciliation process at hospital admission (6). They found that medication accuracy increased from 45.8 to 95% per patient (*p* < 0.001) and medication reconciliation increased from 44.2 to 92.8% (*p* < 0.001) (6). In a randomized prospective study conducted in Norway (201 patients), researchers found that the hospital clinical pharmacists spent significantly less time than the nurses for medication reconciliation (22.9 min for pharmacists and 32.2 min for nurses). Physicians agreed significantly more often with the pharmacist than nurses, which shows who could provide the best possible medication reconciliation process in the hospitals (9). More studies on this topic, including pharmacoeconomic studies, are needed. This would provide better evidence of the hospital clinical pharmacist’s work within the medication reconciliation process. The World Health Organization recommends the important role of the hospital pharmacist in the medication reconciliation process in daily practice and encourages hospital clinical pharmacists to participate in this process (10).

In this context, the main aim of this case report is to describe two clinical cases from the mental health hospital, showcasing the successful collaboration between hospital clinical pharmacists and psychiatrists in the medication reconciliation process.

## Case reports

### Case 1

A psychogeriatric female patient was admitted to Slovenia’s mental health hospital because of anxiety and depression. In addition, the patient suffers from arterial hypertension and heart failure. At the admission, on the patient’s chart, she had the following medications: levothyroxine 75 mg daily, levothyroxine 50 mcg daily (each second day), bisoprolol 1.25 mg daily, esomeprazole 20 mg daily, ursodeoxycholic acid 500 mg daily, mirtazapine 15 mg daily, alprazolam 0.25 mg twice daily, and fixed combination of perindopril, indapamide, and amlodipine 5/1.25/5 mg. The hospital clinical pharmacist provided the best possible medication history and reconciliation within 24 h after admission. He provided the best possible medication history (check the entire medication history through the ePrescribing system and previous discharge papers) in communication with the patient. He recommended to the attending physician rosuvastatin 10 mg initiation because of elevated low-density lipoprotein cholesterol levels (3.72 mmol/L) and the final cardiovascular risk evaluation (Framingham scale). The attending psychiatrist accepted this recommendation. On the day of discharge, the patient’s psychiatrist prepared a discharge paper for her. Hospital clinical pharmacists checked the discharge paper and patient’s chart, and he recognized that levothyroxine 75 mg and the correct dosing of perindopril, indapamide, and amlodipine 5/1.25/5 mg were missing on the discharge paper (compared chart and discharge paper). Hospital clinical pharmacists then reconciled medication with the attending psychiatrist, who accepted both proposals and corrected the discharge paper. The hospital clinical pharmacists then provided a personal medication card to the patient before discharge and explained all the details about pharmacotherapy (gave the card to the patient and included it in the system—each physician in Slovenia would be able to check it immediately in the electronic system). The patient had a good understanding of her medication because the hospital clinical pharmacist discussed medication names, indications, and dosing with her.

### Case 2

A female patient was admitted to the mental health hospital in Slovenia because of bipolar disorder, which has been treated for many years. In addition, she has arterial hypertension, gout, type II diabetes, and heart failure. At the admission, the following medications were listed on the patient’s chart: atorvastatin 40 mg daily, torasemide 5 mg daily, perindopril 4 mg daily, acetylsalicylic acid 100 mg daily, nebivolol 5 mg daily, quetiapine sustained release form 200 mg daily, quetiapine 100 mg daily, and valproic acid 500 mg once daily. The hospital clinical pharmacist provided the best possible medication history and reconciliation within 24 h after hospital admission. He provided the best possible medication history (check the entire medication history through the ePrescribing system and previous discharge papers) in communication with a patient. He recommended to the attending psychiatrist some medication changes (adding omitted medications: metformin 1,000 mg twice daily, linagliptin 5 mg daily, and allopurinol 100 mg daily). The hospital clinical pharmacist recognized the omitted medications by checking the ePrescribing system and in communication with the patient and compared this information with the patient’s chart. The attending psychiatrist accepted these recommendations after a discussion with the hospital clinical pharmacist. On the discharge day, her psychiatrists prepared a discharge paper. The hospital clinical pharmacist checked the discharge paper and patient’s chart, and he did not recognize any medication-related problems (e.g., omissions or medication discrepancies). Hospital clinical pharmacists then provided a personal medication card to the patient before discharge and discussed medication names, indications, and dosing with her and her relatives; thus, the patient understood her medication well.

## Discussion

Elderly patients with mental disorders are often treated with many medications concomitantly (e.g., polypharmacy) and have many medication-related issues during the transition of care, representing an important point for collaboration. These aspects are mostly excluded from randomized controlled trials and meta-analyses, although the topic is highly relevant for daily practice and medication optimization (11, 12). This study adds substantial value to the existing literature by highlighting the significance of hospital clinical pharmacist interventions within the medication reconciliation process in mental health hospitals, a topic not well-described in this part of Europe. These cases may serve as a catalyst for researchers to conduct trials investigating the impact of hospital clinical pharmacists within the medication reconciliation process in mental health hospitals, which can either confirm or challenge our findings.

Clinical pharmacy in psychiatry is included in the treatment processes in some European Countries. This approach is well-known in the United Kingdom and the United States. However, in most European countries, clinical pharmacists are still not regular team members of the ward healthcare team, which was shown in a systematic review published in 2020 (64 studies) (13). The authors found that incorporating psychiatric hospital pharmacist input into interprofessional healthcare teams was the most common pharmacist practice in psychiatric and neurological settings and was associated with significant improvements in patient-level outcomes (13). Oliveira et al. reported positive results of hospital pharmacist-led medication reconciliation in the acute psychiatric ward (14). Their study included 148 admitted patients, and collaboration with psychiatrists was needed in 74% of patients while clarification with psychiatrists was needed in 359 discrepancies (84.12% “drug omission,” 5.57% “drug substitution,” 6.96% “dose change,” and 3.34% “dosage frequency change”) (14). The results show the important role of hospital clinical pharmacists in the medication reconciliation process in a psychiatric hospital. Stuhec and Tement reported that hospital clinical pharmacists’ interventions in the daily rounds in a psychiatric hospital led to fewer medication-related problems, representing an appropriate collaboration with psychiatrists (12). The medication reconciliation process at the transition of care represents a unique opportunity for such collaboration.

These cases show the importance of hospital clinical pharmacist recommendations and their activities within the medication reconciliation process. The first case describes that the inclusion of the hospital clinical pharmacist in the medication reconciliation at the hospital admission can lead to fewer medication-related problems. Hospital pharmacists did not recognize any omitted medications but recognized an untreated condition (hypercholesterolemia), which is in line with the study by Stuhec and Tement, where they showed fewer medication-related problems in inpatients with mental disorders in the ward rounds including clinical pharmacists (12). Comorbidities are frequent in patients with mental disorders, especially elderly patients with schizophrenia. Collaboration with hospital clinical pharmacists can lead to fewer medication-related problems and help psychiatrists deal with comorbidities (e.g., diabetes) (11, 15). This issue was studied in clozapine clinics in the United States. After the clinical pharmacist’s recommendations, the researchers checked the clinical consequences of metabolic and cardiovascular monitoring. The researchers found that pharmacist clinics had statistically higher rates of metabolic and ECG monitoring (glucose 48% vs. 11%, *p* < 0.001; lipids 61% vs. 7.1%, *p* < 0.001; ECG 15% vs. 0%, *p* = 0.001) (15). This study shows that collaboration with hospital clinical pharmacists could lead to better monitoring in patients with clozapine. Hospital pharmacists also could recognize medication-related problems in the discharge paper, which were solved before the patient’s discharge, increasing medication accuracy. This study also highlights the importance of appropriate monitoring, which should be carried out by healthcare professionals at all levels of healthcare. This also emphasizes the need for successful collaboration among pharmacists, physicians, and nurses across various healthcare settings, such as primary care, hospitals, and outpatient clinics. In this context, pharmaceutical services facilitate a smooth transition for patients between primary care and hospitals, which was shown in this study.

The second case shows the important role of hospital clinical pharmacists in medication reconciliation at admission. Omitted medications are seen frequently, and hospital clinical pharmacists can recognize them and recommend medication changes to the attending psychiatrist (16). In this case, the patient with diabetes continued with her medications for diabetes, which were omitted at the admission. In one Slovenian study, which included 108 patients with a median age of 73, 42% of medical records were considered completed medical records. The researchers found that 72.4% of the listed drugs were associated with medication discrepancies. The most discrepancies were often found both in the medication order (76.2%) and discharge letter (69.9%) (16). The authors reported a high rate of discrepancies and the need to implement medication reconciliation, including the participation of hospital clinical pharmacists.

In addition, these cases show the positive impact of this service on the transition of care. From 2023, hospital clinical pharmacists will be doing medication reconciliation in Slovenia. This service is reimbursed (e.g., 50 EUR/patient), meaning that the national insurance company pays extra for each patient in mental health hospitals and can hire clinical pharmacists. Only hospital clinical pharmacist specialists can take over this healthcare program. This process (named seamless care) includes medication reconciliation at admission (including the best possible medication history) and discharge and personal medication card (at the discharge). Hospital clinical pharmacists must be included in the team, have full access to patients and all datasets, and provide medication reviews. All processes have been defined inside this Sub Act (e.g., best possible medication history, medication reconciliation at admission, medication reconciliation at discharge, personal medication card before discharge, and home dispensing) (17, 18).

These case reports are subject to a number of limitations. Clinical outcomes were not measured with any approved scale, and only two cases were included. The following limitation concerns single-hospital clinical pharmacists who provide this service in a psychiatric hospital. To validate these findings, studies employing prospective observational data are required.

## Conclusion

This case study shows that hospital clinical pharmacists could recognize many medication-related problems during the admission and discharge processes, emphasizing the important role of hospital clinical pharmacists in the transition of care in a mental health hospital. Further studies are needed to confirm/reject these findings.

## Data availability statement

The original contributions presented in the study are included in the article/supplementary material, further inquiries can be directed to the corresponding author.

## Ethics statement

Written informed consent was obtained from the individual(s) for the publication of any potentially identifiable images or data included in this article.

## Author contributions

MS: Conceptualization, Writing – original draft, Writing – review & editing. BB: Writing - review & editing.

## References

[1] Ay P Akici A Harmanc H. Drug utilization and potentially inappropriate drug use in elderly residents of a community in Istanbul, Turkey. Int J Clin Pharmacol Ther. (2005) 43:195–202. doi: 10.5414/CPP43000, PMID: 15966466

[2] Vira T Colquhoun M Etchells E. Reconcilable differences: correcting medication errors at hospital admission and discharge. Qual Saf Health Care. (2006) 15:122–6. doi: 10.1136/qshc.2005.015347, PMID: 16585113 PMC2464829

[3] Ryan C Ross S Davey P Duncan EM Francis JJ Fielding S. Prevalence and causes of prescribing errors: the PRescribing outcomes for trainee doctors engaged in clinical training (PROTECT) study. PLoS One. (2014) 9:e79802. doi: 10.1371/journal.pone.0079802, PMID: 24404122 PMC3880263

[4] Santell JP. Reconciliation failures lead to medication errors. Jt Comm J Qual Patient Saf. (2006) 32:225–9. doi: 10.1016/S1553-7250(06)32029-6, PMID: 16649654

[5] Breuker C Macioce V Mura T Castet-Nicolas A Audurier Y Boegner C. Medication errors at hospital admission and discharge: risk factors and impact of medication reconciliation process to improve healthcare. J Patient Saf. (2021) 17:e645–52. doi: 10.1097/PTS.0000000000000420, PMID: 28877049

[6] Smith SB Mango MD. Pharmacy-based medication reconciliation program utilizing pharmacists and technicians: a process improvement initiative. Hosp Pharm. (2013) 48:112–9. doi: 10.1310/hpj4802-112, PMID: 24421448 PMC3839485

[7] Nassaralla CL Naessens JM Chaudhry R Hansen MA Scheitel SM. Implementation of a medication reconciliation process in an ambulatory internal medicine clinic. Qual Saf Health Care. (2007) 16:90–4. doi: 10.1136/qshc.2006.021113, PMID: 17403752 PMC2653166

[8] Aronson J. Medication reconciliation. BMJ. (2017) 356:i5336. doi: 10.1136/bmj.i5336 28087590

[9] Aag T Garcia BH Viktil KK. Should nurses or clinical pharmacists perform medication reconciliation? A randomized controlled trial. Eur J Clin Pharmacol. (2014) 70:1325–32. doi: 10.1007/s00228-014-1741-7 25187339

[10] World Health Organisation. Standard operating protocol assuring medication accuracy at transitions in care. (2016). Available at: http://www.who.int/patientsafety/implementation/solutions/high5s/h5s-sop.pdf

[11] Stuhec M. Antipsychotic treatment in elderly patients on polypharmacy with schizophrenia. Curr Opin Psychiatry. (2022) 35:332–7. doi: 10.1097/YCO.0000000000000808 35788124

[12] Stuhec M Tement V. Positive evidence for clinical pharmacist interventions during interdisciplinary rounding at a psychiatric hospital. Sci Rep. (2021) 11:13641. doi: 10.1038/s41598-021-92909-2 34211019 PMC8249606

[13] Werremeyer A Bostwick J Cobb C Moore TD Park SH Price C. Impact of pharmacists on outcomes for patients with psychiatric or neurologic disorders. Ment Health Clin. (2020) 10:358–80. doi: 10.9740/mhc.2020.11.358 33224694 PMC7653731

[14] Oliveira J Silva TCE Cabral AC Lavrador M Almeida FF Macedo A. Pharmacist-led medication reconciliation on admission to an acute psychiatric hospital unit. Pharm Pract. (2022) 20:2650. doi: 10.18549/PharmPract.2022.2.2650, PMID: 35919807 PMC9296076

[15] Spann G Austin L King E. Pharmacists in clozapine clinics improving physical health monitoring. Ment Health Clin. (2022) 12:193–8. doi: 10.9740/mhc.2022.06.193, PMID: 35801163 PMC9190272

[16] Režonja R Knez L Šuškovič S Košnik M Mrhar A. Comprehensive medication history: the need for the implementation of medication reconciliation processes. Slovenian J Public Health. (2010) 49:202–10. doi: 10.2478/v10152-010-0021-x

[17] Slovenian Pharmacy Act (Pharmacy Practice Act). (2016). Available at: http://www.pisrs.si/Pis.web/pregledPredpisa?id=ZAKO7375 (Accessed December 19, 2022).

[18] Rules on the Provision of Pharmacy Services by Hospital Pharmacies. (2018). Available at: http://pisrs.si/Pis.web/pregledPredpisa?id=PRAV13164 (Accessed December 19, 2022).

